# Detection of IKKε by immunohistochemistry in primary breast cancer: association with EGFR expression and absence of lymph node metastasis

**DOI:** 10.1186/s12885-017-3321-6

**Published:** 2017-05-22

**Authors:** Virginie Williams, Andrée-Anne Grosset, Natalia Zamorano Cuervo, Yves St-Pierre, Marie-Pierre Sylvestre, Louis Gaboury, Nathalie Grandvaux

**Affiliations:** 1 CRCHUM – Centre de recherche du Centre Hospitalier de l’Université de Montréal, 900 rue Saint-Denis, Montréal, Qc H2X 0A9 Canada; 2 Department of Biochemistry and Molecular Medicine, Faculty of Medicine, Université de Montréal, Qc, Montréal, Canada; 3 INRS-Institut Armand-Frappier, INRS, 531 Boul. des Prairies, Laval, Qc H7V 1B7 Canada; 4 IRIC, Université de Montréal, 2900 Boul. Édouard-Montpetit, Montréal, Québec, H3T 1J4 Canada; 5 Department of Social and Preventive Medicine, Ecole de santé publique, Université de Montréal, Qc, Montréal, Canada

**Keywords:** IKKε, Breast, Cancer, EGFR, Metastasis, Immunohistochemistry, Biomarker, Prognosis

## Abstract

**Background:**

IKKε is an oncogenic kinase that was found amplified and overexpressed in a substantial percentage of human breast cancer cell lines and primary tumors using genomic and gene expression analyses. Molecular studies have provided the rational for a key implication of IKKε in breast cancer cells proliferation and invasiveness through the phosphorylation of several substrates.

**Methods:**

Here, we performed immunohistochemical detection of IKKε expression on tissue microarrays constituted of 154 characterized human breast cancer tumors. We further determined the association with multiple clinicopathological parameters and 5-years overall, disease-free and distant disease free survival.

**Results:**

We observed expression of IKKε in 60.4% of the breast cancer tumors. IKKε expression status showed no association with a panel of markers used for molecular classification of the tumors, including ER/PR/HER2 status, or with the molecular subtypes. However, IKKε expression was inversely associated with lymph node metastasis status (*p* = 0.0032). Additionally, we identified a novel association between IKKε and EGFR expression (*p* = 0.0011).

**Conclusions:**

The unexpected observation of an inverse association between IKKε and lymph node metastasis advocates for larger scale immunohistochemical profiling of primary breast tumors to clarify the role of IKKε in metastasis. This study suggests that breast cancer tumors expressing EGFR and IKKε may be potential targets for drugs aiming at inhibiting IKKε activity or expression.

## Background

Breast cancer remains a leading cause of cancer-related mortality in women [1]. Improved outcome and survival of patients have resulted from the identification of Estrogen Receptor (ER), Progesterone Receptor (PR), and HER2 biomarkers that have been used to stratify tumors and define targeted therapies [2]. However, the morphological, clinical and molecular complexity and heterogeneity of breast carcinomas argues for the use of additional specific target genes and pathways as additional biomarkers to define personalized prognostic and predictive therapeutic approach [3]. Initiation and progression of breast cancer relies on the deregulation of a complex network of pathways and genes that control cell proliferation and survival [4]. Knowledge of these pathways provides opportunities for identification of new biomarkers in primary tumors.

The inhibitor of NF-κB kinases (IKK) ε is a member of the IKK family of kinases [5, 6]. IKKε is well recognized for its role in the regulation of distinct NF-κB pathways [7–9] and of the interferon-mediated innate immunity through phosphorylation of Interferon regulatory factors (IRFs) and signal transducer and activator of transcription (STAT) 1 [10–12]. Additional studies have unveiled a key role of IKKε in mammary epithelial cell transformation and invasiveness. Suppression of IKKε by shRNA or transfection of a dominant negative form results in inhibition of anchorage-independent growth and invasiveness of breast cancer cell lines [13, 14]. IKKε-mediated oncogenesis relies on the phosphorylation of multiple substrates, cylindromatosis tumor suppressor (CYLD), estrogen receptor α (ERα), tumor necrosis factor receptor-associated factor 2 (TRAF2), Forkhead box O 3a (FOXO3a) and Akt, and on the regulation of the expression of genes, such as *CCND*1, *MMP-9* and *Bcl-2* [13–19]. In Triple Negative Breast Cancer cells (TNBC), IKKε is involved in the coordinated activation of NF-κB, STAT, and cytokine signaling [20]. IKKε is also involved in the development of resistance to tamoxifen (Tam) treatment. Silencing of IKKε expression sensitizes ER^+^ T47D breast cancer cell line expressing high level of IKKε cells to Tam-induced cell death and apoptosis and to Tam-mediated inhibition of focus formation. Conversely, overexpression of IKKε protects the MCF-7 breast cancer cell line from Tam-induced cell death and apoptosis and reduced Tam-mediated inhibition of focus formation [21].

Analysis of epithelial breast cancer cell lines and primary breast tumors showed copy-number gain or amplification of the 1q32 region resulting in up to 10 copies of the *IKBKE* locus encoding for IKKε [13]. Gene and protein expression studies performed in epithelial breast cancer cell lines, primary breast tumors and in chemically-induced murine mammary breast tumors demonstrated that increased IKKε levels can also result from aberrant expression without gene amplification suggesting that analysis at genomic levels is not appropriate to fully characterize IKKε status in breast cancer [13, 14, 20]. To the best of our knowledge, very limited information is available regarding the relationship between IKKε protein expression and clinicopathological status of primary breast tumors.

Here, we studied IKKε expression by immunohistochemistry (IHC) using tissue microarrays (TMA) of 154 human breast cancer tissues and analyzed the association with clinicopathological parameters and with a panel of biomarkers used for molecular classification of tumors.

## Methods

### Tissue microarrays

High-density tissue microarrays (TMAs) were constructed from formalin-fixed paraffin-embedded material isolated from 154 primary tumor samples and normal adjacent tissues. Tissues were fixed with 10% neutral buffered formalin and paraffin embedded according to usual methods. Samples were cut into 5 μm slices. Three cores were used for each patient. Tumor samples were obtained from patients diagnosed with primary breast cancer at the Centre Hospitalier de l’Université de Montréal. Tumors contained in TMAs were previously characterized on the basis of the histological diagnosis according to the classification of Nottingham modified by Elston and Ellis. The cohort consists of low- and high-grade ductal carcinomas and of medullary carcinomas (typical and atypical). The tumors were previously characterized immunohistochemically for ERα, PR, ErbB2 (Her-2/neu), Ki67 and EGFR among others [22, 23]. Molecular subtypes of patients from the cohort were obtained from the clinical chart and presented the following characteristics: Luminal A: ER^+^/HER2^−^, Ki-67 < 14%; Luminal B: ER^+^/HER2^−^, Ki-67 ≥ 14% or ER^+^/PR^+^/HER2^+^; HER2: ER^−^/PR^−^/HER2^+^; Triple negative: ER^−^/PR^−^/HER2^−^.

### Immunohistochemistry (IHC)

IHC was assessed according to manufacturer recommendations on an immunostainer (Discovery XT system, Ventana Medical Systems, Tucson, AZ). Antigen retrieval was performed with proprietary reagents (cell conditionner 1 for 60mn, Ventana Medical Systems). Monoclonal rabbit anti-IKKε D20G4 (1/50, Cell Signaling #2905) or control Rabbit DA1E mAb IgG XP isotype control (Cell Signaling #3900) antibodies were applied on every sample at room temperature for 4 h. Sections were then incubated with a specific secondary biotinylated antibody for 30 mn. Streptavidin horseradish peroxidase, and 3,3-diaminobenzidine were used according to the manufacturer’s instructions (DABmap detection kit, Ventana Medical Systems). Finally, sections were counterstained with hematoxylin. Each section was scanned at a high resolution (Nanozo-omer, Hammamatsu Photonics K.K.).

### Scoring of IHC staining

IKKε expression was classified according to the following grading system. Two independent observers, including the expert pathologist who made the initial assessment of tissue pathology, scored the intensity of IKKε staining, the percentage of positive cells and the subcellular localization (cytoplasmic and nuclear). IKKε staining intensity and percentage of positive cells were categorized on 0–3 arbitrary scales (Intensity: 0 = absence, 1 = weak, 2 = moderate, 3 = high; Percentage of positive cells: 0 ≤ 1%, 1 ≤ 30%, 2 ≤ 70%, 3 > 70%). The individual categories were multiplied to give an IHC score ranging between 0 and 9 (actual values were 0–4 and 6 and 9) so that the final IHC score reflects the number of cells effectively stained in the tumors tissue and the staining intensity. Localization of IKKε was categorized as cytoplasmic or nuclear. The staining of IKKε corresponds to the mean of staining performed on 3 different cores from a single tumor.

### RNAi transfection and immunoblot

ZR75.1 and MCF7 breast cancer cell lines (obtained from Dr. S. Mader, University of Montreal, Canada) were cultured in RPMI 1640 medium supplemented with 10% heat-inactivated FBS (HI-FBS) and MEM medium supplemented with non-essential amino acids, sodium pyruvate and 10% HI-FBS, respectively. All media and supplements were obtained from Life Technologies. RNAi transfection in ZR75.1 and MCF-7 cells was performed with TransiT siQuest (Mirus) and Dharmafect 1 reagent, respectively, according to the manufacturer’s instructions for 72 h. The sequences of control (siCTRL) and IKKε-specific (siIKKε) RNAi oligonucleotide sequences (Dharmacon) have been previously described [10]. For IKKε immunoblot, cells were lysed as described in [24]. For EGFR immunoblot, cells were lysed by sonication after incubation for 30 min on ice in Triton-X100 lysis buffer (50 mM HEPES pH 7.4, 1 mM EDTA, 250 mM NaCl, 1.5 mM MgCl_2_, 10% Glycerol and 1% Triton-X100) containing 1 mM PMSF, 10 μg/mL aprotinin and 10 μg/mL leupeptin, and quantified using the BCA protein assay (Pierce). Whole cell extracts (WCE), were subjected to SDS-PAGE electrophoresis followed by immunoblot analysis using monoclonal rabbit anti-IKKε D20G4 (1/50, Cell Signaling #2905), monoclonal rabbit anti-EGFR EP38Y (1/4000, Abcam #ab52894) and anti-actin (Chemicon International MAB1501) antibodies. Antibodies were diluted in PBS containing 5% Tween and 5% non-fat dry milk or BSA. Immunoreactive bands were visualized by enhanced chemiluminescence using the Western Lightning Chemiluminescence Reagent Plus (Perkin Elmer Life Sciences) and a CCD-camera LAS400 mini apparatus (GE Healthcare).

### Statistical analysis

Associations between IKKε expression status (positive vs. negative) and both molecular markers and clinicopathological parameters were tested using chi-squared tests. *P*-values <0.05 were considered statistically significant. Kaplan Meier curves were used to estimate overall survival (OS), disease-free survival (DFS) and distant disease free survival (DDFS) over 5 years starting at time of surgery. Survival curves were compared using log-rank test. *P*-values <0.05 were considered statistically significant.

## Results

### Validation of anti-IKKε antibody

**Fig. 1:**
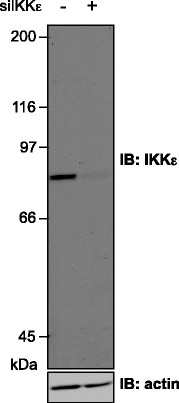
Specificity of the IKKε antibody. ZR75.1 epithelial breast cancer cells expressing high levels of IKKε were transfected with control RNAi (non-targeting) or IKKε-specific RNAi. WCE were resolved by SDS-PAGE and analyzed by immunoblot using anti-IKKε D20G4 and anti-actin antibodies

To study the expression of IKKε at the protein level, the specificity of the commercially available monoclonal rabbit anti-IKKε D20G4 antibody used for TMA IHC staining was first evaluated by immunoblot. WCE derived from the epithelial breast cancer cell line ZR75.1 transfected with control RNAi or RNAi specifically designed to target IKKε were examined (Fig. 1). A single band at 79 kDa corresponding to the expected size of the full length IKKε was detected. This band was dramatically decreased upon transfection of IKKε-specific RNAi, validating the specificity of the antibody and its application for IHC staining of TMA.

### IHC staining of IKKε

**Fig. 2:**
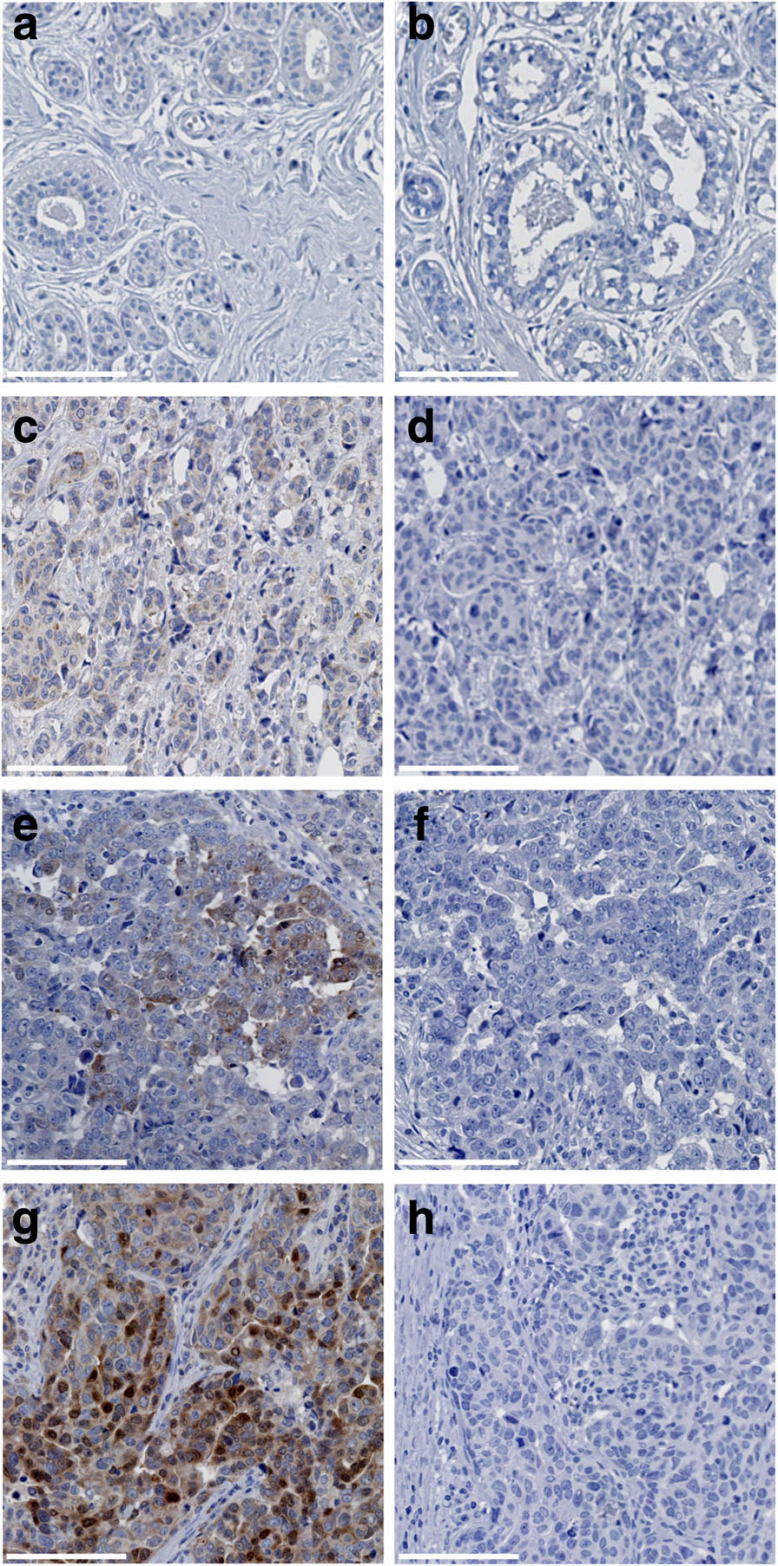
Representative photomicrographs of IKKε immunohistochemical staining of normal mammary gland and tumor breast tissues. Normal breast tissues (**a** and **b**) and breast cancer tissues (**c**–**h**) were stained with rabbit IKKε D20G4 antibody (**a**, **c**, **e** and **g**) or an isotype rabbit control (**b**, **d**, **f** and **h**). Specific IKKε staining was scored on a scale of 0–3. Representative images of intensities 0 (**a**), 1 (**c**), 2 (**e**) and 3 (**g**) are shown. Scale Bars: 100 μm

**Fig. 3:**
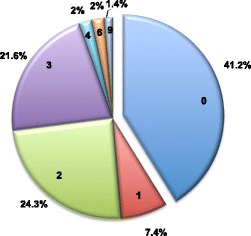
IKKε IHC score distribution. Pie Chart showing the percentage repartition of breast cancer tissues according to the IKKε IHC scores. IKKε IHC score, calculated as detailed in the Methods section, reflects the number of cells effectively stained in the tumors tissue and the staining intensity. IHC score values are 0–4 and 6 and 9

IKKε expression was evaluated on TMA containing triplicates from 154 breast cancer patients and from normal tissues. TMA were stained with the monoclonal rabbit anti-IKKε D20G4 or control rabbit isotype antibodies. Representative IHC photomicrographs are shown in Fig. 2. No significant specific staining was observed with the control rabbit isotype antibody in normal mammary gland or tumor breast tissues (Fig. 2b–h). Normal breast tissues exhibited no detectable IKKε staining (Fig. 2a). Amongst breast tumor samples, 93 out of 154 (60.4%) showed positive IKKε staining of epithelial cancer cells (Fig. 2c–g). In line with previous reports showing IKKε expression in immune cells [5], IKKε was detected both in cancer epithelial cells and invading immune cells in tumor with immune infiltrate (data not shown). An IHC score was determined for 148 out of the 154 samples. Six positive samples had undetermined scores because of discrepancies between the triplicate slides (Fig. 3). The scores of epithelial cells IKKε staining amongst the positive tumors varied significantly, but the vast majority exhibited low (1–3) IHC scores (79/87; 90.8%). As recent reports described substantial nuclear localization of IKKε [25, 26], the localization of IKKε staining in our cohort of breast tumors was carefully assessed. IKKε was restricted to the cytoplasm in the vast majority of tumors. Only 4 tumors out of the 93 positive for IKKε expression exhibited both cytoplasmic and nuclear staining. Although the low number of tumors with nuclear staining prevented correlation studies, it is noteworthy that they were distributed throughout a wide range of IKKε IHC scores. However, the only two tumors with the highest IKKε IHC score of 9 showed cytoplasmic and nuclear staining (Table 1).

**Table 1:** IKKε localization in breast cancer tumors

IKKe IHC score	Cytoplasmic only	Cytoplasmic and nuclear
0	61	0
1	11	0
2	35	1
3	31	1
4	3	0
6	3	0
9	0	2
Total	144	4

### Association between IKKε expression and breast cancer clinicopathological parameters

**Fig. 4:**
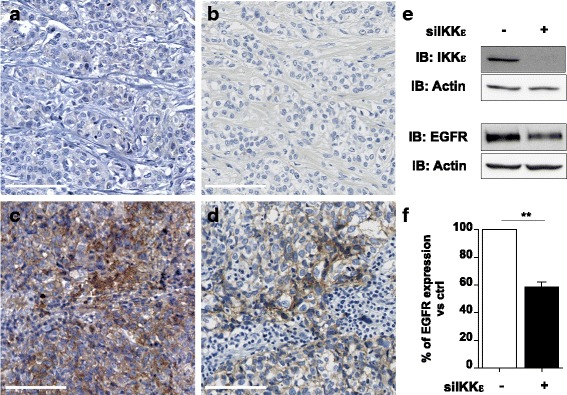
Association between IKKε and EGFR expression. Representative photomicrographs of IKKε (**a**, **c**) and EGFR (**b**, **d**) immunohistochemical staining of tumor breast tissues are shown. Representative images of staining in IKKε^−^/EGFR^−^ (**a** and **b**) and IKKε^+^/EGFR^+^ (**c** and **d**) breast cancer tissues are shown. Scale Bars: 100 μm. In (**e** and **f**), control RNAi (non-targeting) or IKKε-specific RNAi were transfected into MCF-7 cells. Efficiency of IKKε silencing and the impact on EGFR expression were analyzed by immunoblot (IB) using specific antibodies. Actin was used as loading control (**e**). EGFR levels normalized over actin levels were quantified by densitometric analysis using the ImageQuant software. In (**f**), Quantification data are expressed as mean ± SEM from *n* = 4 independent experiments and analyzed using a t-test (**p < 0.01)

The fact that not all patients with breast cancer expressed IKKε suggests that IKKε might be associated with specific characteristic(s). Analysis of the association between IKKε expression and a panel of markers used for molecular classification of tumors and clinicopathological parameters (Table 2) did not reveal significant association with age, specific breast tumor pathological or molecular subtype or expression of the classical markers ER, PR and HER2. Expression of IKKε showed a significant inverse association with the lymph node metastasis status (*p* = 0.0032), as 75.3% of breast tumors expressing IKKε were found negative for lymph node metastasis implicating that IKKε^+^ breast tumors are less likely to present metastasis (Table 2). Amongst the molecular markers tested, epidermal growth factor receptor (EGFR) expression was significantly associated with IKKε expression (*p* = 0.0011), as 72% of the IKKε^−^ breast tumors were also negative for EGFR expression (Table 2 and Fig. 4a–d). To evaluate the possibility that EGFR expression could be placed under the control of IKKε-dependent signalling, the impact of IKKε silencing on EGFR expression levels was tested in MCF-7 epithelial breast cancer cell line. MCF-7 were transfected with control or IKKε-specific RNAi and EGFR expression was monitored by immunoblot. As shown in Fig. 4e and f, silencing of IKKε resulted in the significant inhibition of EGFR expression levels.

**Table 2:** Molecular and clinicopathological parameters correlation according to IKKε expression in breast cancer patients

Parameters		IKKε positive *n* (%)	IKKε negative *n* (%)	*P*-value
Age	<45	11 (11.8)	4 (6.6)	0.4231
	≥45	82 (88.2)	57 (93.4)	
Pathological type	Ductal	63 (67.8)	47 (77)	0.2855
	Medullary	30 (32.3)	14 (23)	
Grade	I	12 (12.9)	3 (4.9)	0.1561
	II	8 (8.6)	3 (4.9)	
	III	73 (78.5)	55 (90.2)	
	unknown	0	0	
Molecular subtypes	Triple negative	54 (58)	40 (65.6)	0.4439
	other	39 (42)	21 (34.4)	
	HER2	7 (7.5)	6 (9.8)	0.8707
	Luminal A	8 (8.6)	8 (13.1)	
	Luminal B	2 (2.2)	3 (4.9)	
	unknown	22	4	
Lymph Node Metastasis	positive	23 (24.7)	30 (49.2)	***0.0032***
	negative	70 (75.3)	31 (50.8)	
	unknown	0	0	
ERα	positive	20 (21.5)	15 (24.6)	0.8872
	negative	70 (75.3)	46 (75.4)	
	unknown	3	0	
PR	positive	13 (14)	6 (9.8)	1
	negative	71 (76.3)	50 (82)	
	unknown	9	1	
HER2	positive	18 (19.4)	6 (9.8)	0.1196
	negative	67 (72)	54 (88.5)	
	unknown	8	1	
EGFR	positive	47 (50.5)	16 (26)	***0.0011***
	negative	38 (40.9)	44 (72)	
	unknown	8	1	
Ki67	positive	83 (89.2)	51 (83.6)	0.1061
	negative	5 (5.4)	9 (14.8)	
	unknown	5	1	
p53	positive	30 (32.2)	10 (16.4)	0.5735
	negative	42 (45.2)	20 (32.8)	
	unknown	21	31	
CK5/6	positive	46 (49.5)	21 (34.4)	0.0568
	negative	42 (45.2)	39 (64)	
	unknown	5	1	
CK14	positive	34 (36.6)	16 (26.2)	0.0869
	negative	48 (51.6)	45 (73.8)	
	unknown	11	0	
GATA3	positive	17 (18.3)	8 (13.1)	0.797
	negative	55 (59.1)	20 (32.8)	
	unknown	21	33	
CD276	positive	37 (39.8)	13 (21.3)	0.3007
	negative	22 (23.7)	14 (23)	
	unknown	34	34	
Galectin 7	positive	38 (40.9)	12 (19.7)	0.4758
	negative	38 (40.9)	18 (29.5)	
	unknown	17	31	

*P*-values were computed using a chi-squared test. Observations with parameter values ‘unknown’ were omitted from the test

*P*-values <0.05 were considered statistically significant and indicated as bold italized

### Prognostic significance of IKKε expression in breast cancer

**Fig. 5:**
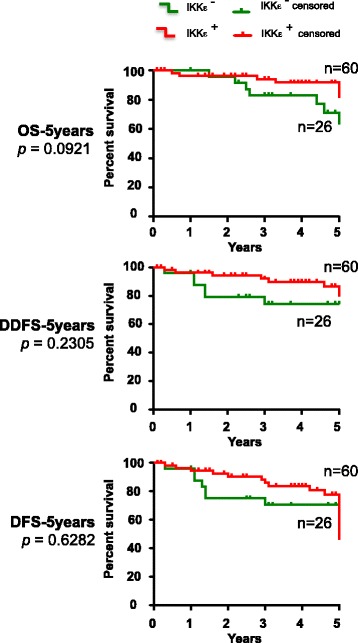
Kaplan-Meier survival curves of breast cancer patients according to IKKε expression. Data correlating the expression of IKKε with the estimates of overall survival (OS), disease free survival (DFS) and distant disease free survival (DDFS) in breast cancer patients over 5 years after surgery are shown. A log-rank test was used to calculate statistical significance

To further assess the clinical relevance of IKKε expression in breast tumors, we analyzed the 5-year after surgery overall survival (OS), disease-free survival (DFS) and distant disease free survival (DDFS) follow-up information available from IKKε negative (*n* = 26) and IKKε positive (*n* = 60) tumors (Fig. 5). Comparison of survival curves by log-rank test in Kaplan-Meier survival analyses showed that patients in the IKKε^+^ subgroup exhibit no significant differences of OS, DFS or DDFS compared to IKKε^−^ subgroup.

## Discussion

The implication of IKKε in breast cancer tumorigenesis provides opportunities for targeted therapies. However, the relationship between IKKε expression in primary breast carcinomas and clinicopathological markers remained to be established. Previous genomic and gene expression analyses have highlighted increased IKKε expression, accompanied or not by gene amplification, in 30% of breast tumors [13, 14, 20]. This same percentage was also observed by IHC staining of a very limited number of primary breast tumors (*n* = 20) [13]. Here, IHC staining of 154 tumor breast tissues revealed a substantially higher percentage (60.4%) of tumor exhibiting IKKε protein expression. The vast majority of breast tumors in our cohort exhibited low staining levels (IHC score: 1–3) and the detection was restricted to the cytoplasm. In contrast to previous reports showing prominent nuclear staining of IKKε in human prostate cancer and Kaposi sarcoma tumors [25, 26], only 4 out of the 93 IKKε^+^ tumors showed localization of IKKε in the nucleus. The molecular mechanisms and functional significance of the nuclear localization of IKKε still remains elusive, but would benefit from further molecular studies and larger scale correlation analyses.

Corroborating previous observations made by gene expression studies [13, 20], we failed to find an association with tumor subtypes or with ER/PR/HER2 status. However, IKKε protein expression exhibited a significant inverse association with lymph node metastasis. This result differs from the absence of correlation previously observed between *IKBKE* copy-number gain and the presence of lymph node involvement at diagnosis [13]. However, in the latter study, only 30 breast tumor specimens were analyzed and thus the lack of correlation may be due to the small number of specimens studied. The discrepancy with our study might also reflect the previous observation that IKKε levels in breast cancer does not solely results from gene amplification, but also occurs as a result of aberrant expression due to yet to be fully characterized mechanisms [13, 14, 20]. Thus, breast tumors harboring *IKBKE* copy-number gain studied by Boehm and coll. represent only a subset of IKKε-positive tumors [13]. The inverse association between IKKε and lymph node metastasis was unexpected considering previously published molecular studies performed in cell lines, which were paradoxically suggestive of a potential association between IKKε expression and invasiveness. Indeed, silencing of IKKε or expression of a dominant negative form of IKKε in the SK-BR-3 or NF639 breast cancer cell lines resulted in a defect of cell migration and invasion abilities, two properties essential for the spreading of cancer cells and metastasis [14, 18]. Although these in vitro assays performed using single cell types provide information regarding cell autonomous mechanisms contributing to metastasis, they do not take into account the in vivo microenvironment of the tumor, which is also important in the metastatic process. Further in vivo mechanistic studies will be required to clarify the role of IKKε in the metastasis process. Additionally, larger scale IHC profiling of primary breast tumors including TNM stage classification will allow assessment of the association with distant metastases that were not evaluated in our cohort.

To the best of our knowledge our study is the first to report an association between IKKε and expression of the EGFR marker in breast tumors. The observation that silencing of IKKε in epithelial breast cancer cells significantly diminishes EGFR expression levels, suggest that the association between IKKε and EGFR might result at least in part from EGFR expression levels being placed under the control of IKKε-dependent signalling. EGFR is a tyrosine kinase receptor in the HER family, which is either overexpressed or mutated in breast cancer cells [27, 28] and is involved in cancer pathogenesis and progression [29]. EGFR is overexpressed in all subtypes of breast cancer, but is more frequently associated with aggressive TNBC and inflammatory breast tumors [30, 31]. Here, we did not observe an association between IKKε and TNBC. Several EGFR-targeting therapies have been developed, but have shown limited benefit and resistance has been observed, leading to the search of additional biomarkers that could be targeted simultaneously [32, 33]. Interestingly, a functional relationship between IKKε and EGFR has also been described in the context of non-small lung cancer cells harboring activating EGFR mutations [34]. EGFR directly interacts with and phosphorylates IKKε leading to activation of downstream Akt pathway. Silencing of IKKε or treatment with the IKKε inhibitor amlexanox selectively decreased cell survival, providing rational support to target IKKε as a therapeutic strategy for non-small lung cancer [34]. Our observation warrants further studies to determine the functional relationship between IKKε and EGFR in breast cancer. Particularly, mechanistic studies will be necessary to determine how IKKε-dependent signaling cascade(s) contributes to EGFR expression and if IKKε expression correlates with mutated EGFR. Alternatively, based on the positive association between IKKε and EGFR expression it would also be interesting to determine whether EGFR activation is required for IKKε expression in IKKε^+^/EGFR^+^. This knowledge will help determine if therapeutic strategies targeting IKKε are relevant for IKKε^+^/EGFR^+^ breast tumors.

In ovarian cancer, IKKε expression was found to be a relatively strong predictor of poor clinical outcome [35]. In contrary, expression of IKKε and the closely related kinase TBK1 in gastric cancer was not associated with difference in survival when compared to IKKε^−^/TBK1^−^ subgroup [36]. Although Kaplan-Meier curves show a tendency of IKK^+^ subgroup to have a better OS, analysis of our cohort did not show statistically significant relationship between IKKε expression and the clinical outcome. However, we cannot exclude that the absence of statistical significance could be due to a limitation of our follow-up study in term of number of patients examined and low number of events in the subgroups. Therefore, additional studies including larger cohort of patients will be required to verify the association of IKKε expression with breast cancer prognosis.

Compelling evidence of the involvement of IKKε in the pathophysiology of breast cancer and other diseases provided the rational for the search of therapeutic inhibitor of IKKε [37]. IKKε and TBK1 share an overall 65% sequence similarity and 72% identity in the kinase domain [38]. A series of dual TBK1/IKKε inhibitor compounds have been identified with relative specificities amongst other kinase [37, 39–42]. Some of these compounds exhibit antiproliferative activity in breast cancer cell lines [42]. Recently, the antiallergic small molecule amlexanox was found to be a selective inhibitor of TBK1/IKKε [43, 44]. Additionally, we showed that the redox-regulating compounds triphenylmethane dyes, Gentian Violet and Brilliant Green, and nitroxide Tempol inhibit IKKε, but not TBK1, expression in breast cancer cells [24]. This finding offers alternative therapeutic avenue to target IKKε in breast cancer.

## Conclusions

Immunohistochemical analysis of IKKε expression in our cohort of primary breast tumors revealed an unexpected inverse association with lymph node metastasis and a positive association with EGFR status. Both observations advocate additional studies, including larger scale IHC profiling of primary breast tumors, to determine the functional relationship between EGFR and IKKε and importantly to clarify the role of IKKε in metastasis. Additionally, these studies will be essential to confirm if IKKε can be used as a biomarker to define personalized prognostic and the potential of targeting IKKε for therapeutic opportunities, particularly in EGFR+ breast tumors.

## Abbreviations

CYLD: Cylindromatosis tumor suppressor
DDFS: Distant disease free survival
DFS: Disease-free survival
EGFR: Epidermal growth factor receptor
ER: Estrogen Receptor
FBS: Fetal bovine serum
FOXO3a: Forkhead box O 3a
HI: Heat-inactivated
IHC: Immunohistochemistry
IKK: Inhibitor of NF-κB kinase
IRF: Interferon regulatory factor
OS: Overall survival
PR: Progesterone receptor
STAT: Signal transducer and activator of transcription
Tam: Tamoxifen
TBNC: Triple negative breast cancer cell
TMA: Tissue microarrays
TRAF: Tumor necrosis factor receptor-associated factor
WCE: Whole cell extracts
